# Artificial Intelligence and Machine Learning in Predicting the Response to Immunotherapy in Non-small Cell Lung Carcinoma: A Systematic Review

**DOI:** 10.7759/cureus.61220

**Published:** 2024-05-28

**Authors:** Tanya Sinha, Aiman Khan, Manahil Awan, Syed Faqeer Hussain Bokhari, Khawar Ali, Maaz Amir, Aneesh N Jadhav, Danyal Bakht, Sai Teja Puli, Mohammad Burhanuddin

**Affiliations:** 1 Internal Medicine, Tribhuvan University, Kathmandu, NPL; 2 Medicine, Liaquat College of Medicine and Dentistry, Karachi, PAK; 3 General Practice, Liaquat National Hospital and Medical College, Karachi, PAK; 4 Surgery, King Edward Medical University, Lahore, PAK; 5 Medicine and Surgery, King Edward Medical University, Lahore, PAK; 6 Pediatrics, Bharat Ratna Dr. Babasaheb Ambedkar Memorial Hospital, Mumbai, IND; 7 Medicine and Surgery, Mayo Hospital, Lahore, PAK; 8 Internal Medicine, Bhaskar Medical College, Hyderabad, IND; 9 Medicine, Bhaskar Medical College, Hyderabad, IND

**Keywords:** clinical variables, genomic data, radiomics, deep learning, nsclc, non-small cell lung carcinoma, immunotherapy response, predictive biomarkers, machine learning, artificial intelligence

## Abstract

Non-small cell lung carcinoma (NSCLC) is a prevalent and aggressive form of lung cancer, with a poor prognosis for metastatic disease. Immunotherapy, particularly immune checkpoint inhibitors (ICIs), has revolutionized the management of NSCLC, but response rates are highly variable. Identifying reliable predictive biomarkers is crucial to optimize patient selection and treatment outcomes. This systematic review aimed to evaluate the current state of artificial intelligence (AI) and machine learning (ML) applications in predicting the response to immunotherapy in NSCLC. A comprehensive literature search identified 19 studies that met the inclusion criteria. The studies employed diverse AI/ML techniques, including deep learning, artificial neural networks, support vector machines, and gradient boosting methods, applied to various data modalities such as medical imaging, genomic data, clinical variables, and immunohistochemical markers. Several studies demonstrated the ability of AI/ML models to accurately predict immunotherapy response, progression-free survival, and overall survival in NSCLC patients. However, challenges remain in data availability, quality, and interpretability of these models. Efforts have been made to develop interpretable AI/ML techniques, but further research is needed to improve transparency and explainability. Additionally, translating AI/ML models from research settings to clinical practice poses challenges related to regulatory approval, data privacy, and integration into existing healthcare systems. Nonetheless, the successful implementation of AI/ML models could enable personalized treatment strategies, improve treatment outcomes, and reduce unnecessary toxicities and healthcare costs associated with ineffective treatments.

## Introduction and background

Non-small cell lung carcinoma (NSCLC) is a prevalent and aggressive form of lung cancer that accounts for approximately 85% of all lung cancer cases [1]. Despite advances in treatment modalities, the prognosis for NSCLC remains poor, with a five-year survival rate of less than 20% for metastatic disease [2]. Immunotherapy, particularly immune checkpoint inhibitors (ICIs), has emerged as a promising treatment option, revolutionizing the management of NSCLC. However, the response rates to immunotherapy are highly variable, highlighting the need for predictive biomarkers to identify patients most likely to benefit from these therapies [3]. Immunotherapy in NSCLC aims to harness the immune system to recognize and eliminate cancer cells. ICIs, such as anti-programmed cell death protein 1 (PD-1) and anti-programmed death-ligand 1 (PD-L1) antibodies, have demonstrated remarkable clinical efficacy in a subset of NSCLC patients [4]. These therapies work by blocking the inhibitory signals that suppress the immune system's ability to recognize and attack cancer cells. However, not all patients respond favorably to immunotherapy, and some experience significant toxicities. Therefore, identifying reliable predictive biomarkers is crucial to optimizing patient selection and treatment outcomes. Predictive biomarkers, such as PD-L1 expression, tumor mutational burden (TMB), and immune cell infiltration, have been explored to guide immunotherapy decisions in NSCLC. However, these biomarkers have limitations and do not fully capture the complex interplay between the tumor and the immune system [5,6]. This has led to the exploration of artificial intelligence (AI) and machine learning (ML) techniques to integrate multiple biomarkers and clinical factors for more accurate prediction of immunotherapy responses.

AI and ML are powerful computational approaches that can analyze large and complex datasets to identify patterns and make predictions. In the context of NSCLC immunotherapy, AI and ML algorithms can be trained on various data sources, including genomic, transcriptomic, radiologic, and clinical data, to develop predictive models [7]. These models have the potential to better stratify patients based on their likelihood of response to immunotherapy, ultimately improving treatment outcomes and minimizing unnecessary toxicities. The rationale for this systematic review is to comprehensively evaluate the current state of AI and ML applications in predicting the response to immunotherapy in NSCLC. By synthesizing the available evidence, this review aims to provide insights into the most promising approaches, identify gaps in knowledge, and guide future research in this rapidly evolving field. The primary objectives of this systematic review are to assess the performance and predictive ability of AI and ML models in forecasting the response to immunotherapy in NSCLC. This review also aims to identify the key features and biomarkers that contribute to the accurate prediction of immunotherapy response in NSCLC. Additionally, it also provides recommendations for future research and clinical implementation of these predictive models.

## Review

Materials and methods

This systematic review follows the guidelines outlined in the Preferred Reporting Items for Systematic Reviews and Meta-Analyses (PRISMA) to ensure a methodical and thorough assessment of studies exploring the role of AI and ML in predicting the response to immunotherapy in NSCLC.

Search Strategy

A systematic search strategy was conducted across major electronic databases, including PubMed, Embase, Hinari, and the Cochrane Library. The search strategy combined Medical Subject Headings (MeSH) terms and keywords relevant to artificial intelligence, machine learning, immunotherapy, and NSCLC. Boolean operators (AND, OR) were used to refine the search and identify studies meeting predetermined inclusion criteria. For example, the search strategy (("Machine Learning" OR "Artificial Intelligence") AND "Immunotherapy" AND ("Lung Neoplasms" OR "Lung Cancer") AND ("Predict*" OR "Response" OR "Treatment Outcome")) was used to identify relevant articles.

Eligibility Criteria

To ensure the inclusion of high-quality and pertinent studies, strict eligibility criteria were established. Included studies were required to investigate the predictive capabilities of AI/ML in the context of immunotherapy responses, specifically in NSCLC. Only studies published in peer-reviewed journals up to February 2024 were considered. Studies lacking sufficient data on AI/ML applications in NSCLC immunotherapy were excluded. Additionally, studies focusing solely on animal models or published in languages other than English, as well as those without full-text availability, were excluded to maintain accessibility and comprehensibility.

Data Extraction and Synthesis

Two independent reviewers conducted an initial screening of titles and abstracts, followed by a detailed assessment of full texts to ensure alignment with the inclusion criteria. Any discrepancies between reviewers were resolved through discussion or consultation with a third reviewer. Relevant data, including study characteristics, AI/ML methodologies employed, and outcomes related to immunotherapy response prediction, were systematically extracted using a predefined data extraction form.

Data Analysis

Due to the expected diversity in study designs and outcome measures, a narrative synthesis approach was employed. This involved identifying key themes and patterns in the literature regarding the predictive role of AI/ML in NSCLC immunotherapy response. This method ensures a comprehensive and transparent evaluation of the available evidence.

This meticulous methodology serves as the framework for systematically reviewing and synthesizing evidence on the predictive capabilities of AI/ML in NSCLC immunotherapy response.

Results

Study Selection Process

**Figure 1:**
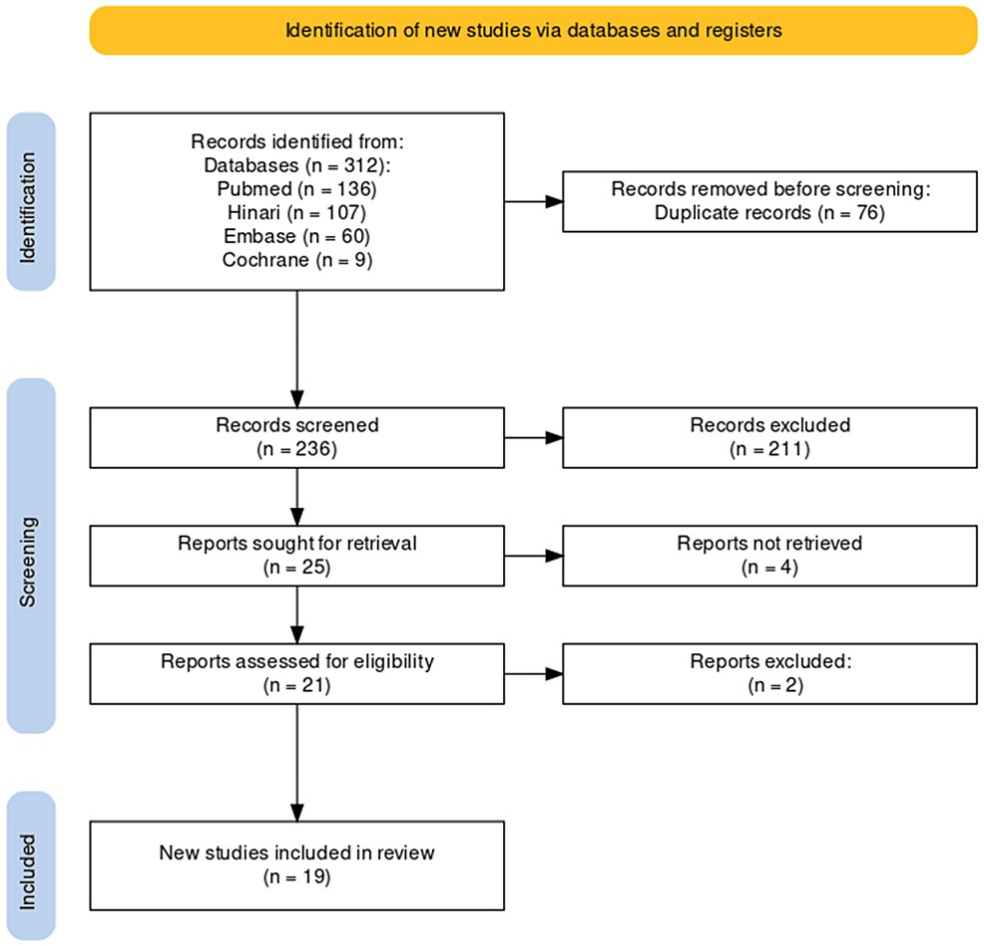
PRISMA diagram illustrating the study selection process. PRISMA: Preferred Reporting Items for Systematic Reviews and Meta-Analyses

Consistent with the PRISMA guidelines, the study selection process was rigorously executed to ensure transparency and methodological rigor. A comprehensive search initially yielded a total of 312 studies. Following the removal of duplicates, 236 unique studies remained. Screening of titles and abstracts led to the exclusion of 211 records that did not meet predefined relevance criteria. Subsequent evaluation of the full texts of the remaining articles resulted in the exclusion of six studies that did not align with stringent inclusion criteria. Following this stringent selection process, 19 studies were identified as suitable for inclusion in the systematic review. The study selection process is illustrated in the PRISMA flowchart (Figure 1).

Study Characteristics

All the studies included in this systematic review were retrospective cohorts conducted in various countries. Eight studies were from China, four from the USA, two each from Italy and Canada, and one each from Denmark, France, and Germany. The sample sizes of the included studies varied considerably, ranging from relatively small cohorts of around 100 patients to larger cohorts exceeding 8900 patients. This diversity in sample sizes allows for an evaluation of the robustness and generalizability of the AI and ML models across different population sizes and settings (Table 1).

**Table 1:** Study characteristics of included studies.

Author	Year	Country	Study design	Sample size
Li et al. [8]	2024	China	Retrospective cohort	136
Yolchuyeva et al. [9]	2024	Canada	Retrospective cohort	149
Yolchuyeva et al. [10]	2023	Canada	Retrospective cohort	223
Wei et al. [11]	2023	Japan	Retrospective cohort	Cohort 1: 123 Cohort 2: 99
Vanguri et al. [12]	2022	USA	Retrospective cohort	247
He et al. [13]	2022	China	Retrospective cohort	236
Prelaj et al. [14]	2022	Italy	Retrospective cohort	164
Li et al. [15]	2022	China	Retrospective cohort	289
Liu et al. [16]	2022	China	Retrospective cohort	853
Prelaj et al. [17]	2022	Italy	Retrospective cohort	480
Wang et al. [18]	2022	China	Retrospective cohort	162
Peng et al. [19]	2022	China	Retrospective cohort	915
Trebeschi et al. [20]	2021	Denmark	Retrospective cohort	152
Benzekry et al. [21]	2021	France	Retrospective cohort	298
Yang et al. [22]	2021	China	Retrospective cohort	200
Arbour et al. [23]	2021	USA	Retrospective cohort	453
He et al. [24]	2020	China	Retrospective cohort	327
Khorrami et al. [25]	2020	USA	Retrospective cohort	139
Siah et al. [26]	2019	USA	Retrospective cohort	8,925

The main findings of the included studies are summarized in the following table (Table 2).

**Table 2:** Summary of the main findings of the studies included in this systematic review. AI: artificial intelligence; ML: machine learning; DL: deep learning; PD-L1: programmed death-ligand 1; PET/CT: positron emission tomography/computed tomography; PFS: progression-free survival; OS: overall survival; C-index: concordance index; KNN: k-nearest neighborhood; RL: ReliefF; XGBoost: extreme gradient boosting; MI: mutual information; CIRI: cytokine-based ICI response index; TMB: tumor mutational burden; RECIST: response evaluation criteria in solid tumors; DCR: disease control rate; ORR: overall response rate; OS6: 6-month overall survival; OS24: 24-month overall survival; PFS3: 3-month progression-free survival; TTF3: 3-month time to treatment failure; ITH: intratumor heterogeneity; HLA LOH: human leukocyte antigen loss of heterozygosity; pDCB: predicted durable clinical benefit; TMV: tumor mutation volume; CNN: convolutional neural network; SVM: support vector machine; RF: random forest; TMBRB: tumor mutational burden radiomics biomarker; DelRADx: delta-radiomics; LDA: linear discriminant analysis; TIL: tumor-infiltrating lymphocyte; ICI: immune checkpoint inhibitor; OR: objective response; XAI: explainable AI

Author	Year	Clinical objectives	Variables	Results/main findings
Li et al. [8]	2024	Predict PD-L1 expression	PET/CT-based deep learning radiomics model	PET/CT-based deep learning radiomics model can accurately predict PD-L1 expression in NSCLC.
Yolchuyeva et al. [9]	2024	PFS, OS	C-index, KNN with ReliefF (RL) feature selection for PFS. XGBoost with MI feature selection for OS.	Appropriate feature selection method combined with an ML approach to develop clinically usable prognostic models for patients treated with ICIs in a first-line setting.
Yolchuyeva et al. [10]	2023	OS in response to anti-PD-1/PD-L1 immunotherapy	CIRI	Parsimonious survival risk models in NSCLC patients treated with immunotherapy, particularly in identifying short- and long-term survivors. This finding may enable clinicians to design more effective therapeutic regimens or modify treatment strategies for the group of short-term survivors.
Wei et al. [11]	2023	OS in response to anti-PD-1/PD-L1 immunotherapy	CIRI, peripheral blood cytokine profiles	The CIRI model is highly accurate and reproducible in determining the patients with NSCLC who would benefit from anti-PD-1/PD-L1 therapy with prolonged OS and may aid in clinical decision-making before and/or at the early stage of treatment.
Vanguri et al. [12]	2022	To predict immunotherapy response using expert-guided ML	Integrating medical imaging, histopathologic, and genomic features including computed tomography scan images, and digitized PD L1 immunohistochemistry slides.	ML to integrate features from computed tomography scan images, PD-L1 immunohistochemistry, and genomics into a multimodal predictor of response to anti-PD-L1 in patients with NSCLC that outperformed single features such as tumor mutational burden.
He et al. [13]	2022	To predict the efficacy of ICI monotherapy in patients with advanced NSCLC.	PFS, OS, OSRS and PFSRS	A CT imaging-based score with the potential to become an independent prognostic factor to screen patients who would benefit from ICI treatment, which suggested that CT radiomics could be applied for individualized immunotherapy of NSCLC.
Prelaj et al. [14]	2022	Aimed at using AI and ML tools to improve response and efficacy predictions in aNSCLC patients treated with IO.	OS	Development of an ML algorithm based on real-world data, explained by SHAP techniques, and able to accurately predict the efficacy of immunotherapy in sets of NSCLC patients.
Li et al. [15]	2022	The efficacy evaluation of immunotherapy to lung squamous carcinoma patients, especially the DCR and ORR models	DCR, ORR, PFS, and OS	DCR model demonstrated robust performance both internally and externally, with high AUC values, indicating its effectiveness in evaluating immunotherapy efficacy. Similarly, ORR, PFS, and OS models also exhibited strong predictive capabilities, albeit with slightly lower AUC values in external validation.
Liu et al. [16]	2022	The value of the derived ML signature on immunotherapy efficacy was evaluated and compared with the TMB and other clinical characteristics	predictive genes	Fewer genetic tests are sufficient to predict immunotherapy efficacy, we used ML to screen out gene panels, which are used to calculate TMB. Therefore, we obtained the 88-gene panel, which showed a favorable prediction performance and stratification effect compared to the original TMB.
Prelaj et al. [17]	2022	Efficacy of immunotherapy using eXplainable AI (XAI) and ML	DCR, ORR, OS6 and OS24, PFS3 and TTF3	XAI and ML predict immunotherapy efficacy in advanced NSCLC patients, achieving high accuracy for outcomes such as DCR, OS6, and TTF, with features like neutrophil to lymphocyte ratio strongly influencing predictions. Additionally, the model identified the importance of factors like performance status and PD-L1 expression in predicting treatment response, aiding in personalized treatment decisions.
Wang et al. [18]	2022	Robust predictive model to predict durable response to ICIs in NSCLC patients based on multiple genomic features	TMB, ITH, and HLA LOH	A multi-feature model that can effectively predict the efficacy of NSCLC patients treated with ICIs, which can help in clinical decision-making. In addition, patients with pDCB could be considered as more suitable candidates for treatment with ICIs
Peng et al. [19]	2022	Estimate clinical benefit in patients with NSCLC before immunotherapy	PD-L1, TMV, PFS, OS	Combining three cML methods (CNN, SVM, and RF) yielded a robust comprehensive nomogram for predicting PFS and OS in the three cohorts (each p < 0.001). The proposed DL method based on mutational genes revealed the potential value of clinical benefit prediction in patients with NSCLC and provides novel insights for combined ML in PD-1/PD-L1 blockade.
Trebeschi et al. [20]	2021	Identify morphological changes on chest CT	CT	The results demonstrate that DL can quantify tumor- and non-tumor-related morphological changes important for prognostication on serial imaging.
Benzekry et al. [21]	2021	Predict response to ICIs	DCR, PFS,OS	Pre-ICI blood counts and clinical status predicted better DCR. ML utilized these associations for individual treatment response prediction, suggesting potential improvements with more variables and independent cohort validation.
Yang et al. [22]	2021	Integrate multimodal serial information from CT with laboratory and baseline clinical information and predict response to PD L1 inhibitors	PFS, OS, CT, blood samples	The patients were divided into high- and low-risk non-responders using the model. The low-risk group had significantly longer progression-free survival than the high-risk group. The SimTA-based multi-omics serial deep learning provides a promising methodology for predicting the response of advanced NSCLC patients to anti-PD-1/PD-L1 monotherapy.
Arbour et al. [23]	2021	Estimate gold-standard RECIST in patients with NSCLC Treated with PD-1 Blockade	RECIST, radiomics	In addition to determining the best overall response, the model accurately evaluated the occurrence of progression and date of progression, enabling assessment of RECIST PFS. Response assessments predicted by the DL model show close similarity to RECIST response categorization with respect to the long-term impact on overall survival.
He et al. [24]	2020	Correlation between DL radiomic biomarker and TMB, including its predictive value for ICI treatment response	OS, PFS, CT, TMB	TMBRB divided patients into high- and low-risk groups with distinct survival outcomes. Using DL and CT images, researchers created a non-invasive biomarker to differentiate High-TMB from Low-TMB, aiding in decision-making for ICIs in advanced NSCLC.
Khorrami et al. [25]	2020	OS and Response to Immunotherapy	DRS, OS	The study examined the association between DRS and OS. Additionally, it evaluated the correlation of DelRADx features with TIL density in diagnostic biopsies (n=36). Using the LDA classifier, it achieved high AUCs in distinguishing responders from nonresponders. DRS showed a significant association with OS.
Siah et al. [26]	2019	ML models for OR, PFS, and OS points in patients with advanced NSCLC	OR, OS, PFS	The models showed strong predictive performance for OR, PFS, and OS. Calibration plots indicated good agreement between actual and predicted survival probabilities. Kaplan-Meier survival curves revealed significant differences in survival between low- and high-risk groups for both PFS and OS (log-rank test, p < 0.001).

Discussion

AI and ML algorithms have gained significant traction in medical applications due to their ability to analyze vast amounts of data, identify intricate patterns, and make accurate predictions. These techniques have been employed in various areas, such as medical imaging analysis, disease diagnosis, drug discovery, and personalized treatment planning [27]. In oncology, AI/ML approaches have been utilized for tumor detection, segmentation, and staging, as well as for predicting treatment outcomes and survival [28]. The application of AI and ML techniques in oncology has been extensively explored, particularly in the realm of precision medicine. These algorithms have been employed to analyze multi-omics data, including genomics, transcriptomics, and proteomics, to identify biomarkers and pathways associated with cancer development, progression, and response to therapy [29]. Additionally, AI/ML models have been developed to integrate clinical, molecular, and imaging data, enabling more personalized and tailored treatment strategies.

The studies included in this review aimed to develop and validate AI/ML models for predicting immunotherapy response, progression-free survival (PFS), and overall survival (OS) in NSCLC patients receiving ICIs [9,13,14,19,25,26]. The studies utilized diverse AI/ML techniques, including deep learning (DL), artificial neural networks (ANNs), support vector machines (SVMs), random forests (RFs), and gradient boosting methods (e.g., XGBoost). These algorithms were applied to various data modalities, such as medical imaging (computed tomography (CT), positron emission tomography (PET)), genomic data (TMB, gene expression), clinical variables (performance status, blood counts), and immunohistochemical markers (PD-L1, TILs) [8-26]. The studies employed various performance metrics to evaluate the predictive accuracy of their AI/ML models. Common metrics included area under the receiver operating characteristic curve, concordance index, sensitivity, specificity, positive predictive value, and negative predictive value. Siah et al. also reported calibration plots and Kaplan-Meier survival curves to assess the agreement between predicted and observed outcomes [26].

Several studies have demonstrated the ability of AI and ML models to accurately predict immunotherapy response, PFS, and OS in NSCLC patients. For instance, Li et al. (2024) reported that their deep-learning radiomics model could accurately predict PD-L1 expression, a key biomarker for immunotherapy response [8]. Similarly, Yolchuyeva et al. (2023, 2024) developed ML models incorporating cytokine profiles and clinical variables that effectively predicted OS and PFS in patients receiving anti-PD-1/PD-L1 immunotherapy [9,10]. Vanguri et al. (2022) and He et al. (2022) utilized multimodal data, including CT images, genomic features, and clinical variables, to build ML models that outperformed single biomarkers like TMB in predicting immunotherapy response [12,13]. Prelaj et al. (2022) employed eXplainable AI (XAI) and ML techniques to develop a model that accurately predicted disease control rate, OS, and time to treatment failure, while also identifying influential features such as neutrophil-to-lymphocyte ratio and PD-L1 expression [14,17]. Several studies, including those by Li et al. (2022), Liu et al. (2022), Wang et al. (2022), and Peng et al. (2022), leveraged genomic data and ML algorithms to predict immunotherapy response and survival outcomes, often outperforming traditional biomarkers like TMB [15,16,18,19]. Importantly, some studies, such as those by Trebeschi et al. (2021), Benzekry et al. (2021), Yang et al. (2021), and Arbour et al. (2021), employed ML techniques to analyze serial imaging data, laboratory values, and clinical information, demonstrating the potential of these approaches in monitoring treatment response and predicting long-term outcomes [20-23].

One of the significant challenges in developing AI/ML models for predicting immunotherapy responses is the availability and quality of the data. Many studies relied on retrospective data from clinical trials or single-center cohorts, which may introduce selection bias and limit the generalizability of the findings. Additionally, the heterogeneity in data collection, processing, and annotation across different centers and studies can pose challenges in integrating and harmonizing data for model development and validation. While AI/ML models have demonstrated exceptional predictive performance, their "black box" nature can make it challenging to understand the underlying decision-making process. Interpreting the relationships between input features and model predictions is crucial for gaining clinical insights and ensuring trust in these systems [30]. Efforts have been made to develop interpretable AI/ML techniques, such as XAI and feature importance analysis (e.g., SHAP values), but further research is needed to improve the transparency and explainability of these models [31].

Translating AI/ML models from research settings to clinical practice poses significant challenges. Issues related to regulatory approval, data privacy, and security, as well as the integration of these models into existing healthcare systems and clinical workflows, need to be addressed [32]. Additionally, ensuring the generalizability and robustness of AI/ML models across diverse patient populations and healthcare settings is crucial for their successful implementation. The deployment of AI/ML systems in healthcare raises ethical concerns related to data privacy, algorithmic bias, and the potential for perpetuating or amplifying existing disparities in healthcare access and quality [33]. It is essential to address these ethical issues and develop robust frameworks for the responsible development and deployment of AI/ML technologies in clinical settings.

As AI/ML techniques continue to evolve, novel algorithms and architectures may offer improved predictive performance and interpretability. For example, advances in areas such as federated learning, transfer learning, and multi-task learning could enable more efficient model development and deployment across different healthcare systems and patient populations [34]. Additionally, the integration of causal reasoning and XAI techniques could enhance the transparency and trustworthiness of AI/ML models in clinical decision-making. While AI/ML models have shown promising results in predicting immunotherapy response, their integration with traditional biomarkers, such as PD-L1 expression, TMB, and TILs, could potentially improve their predictive accuracy and clinical utility. Multimodal approaches that combine molecular, clinical, and imaging data may provide a more comprehensive understanding of the complex factors influencing immunotherapy response and enable personalized treatment strategies.

The ultimate goal of AI/ML applications in oncology is to enable truly personalized medicine, where treatment decisions are tailored to individual patients based on their unique molecular, clinical, and imaging profiles [35]. By integrating diverse data sources and leveraging the predictive power of AI/ML algorithms, it may be possible to identify subgroups of patients who are most likely to respond to specific immunotherapies or combination treatments, thereby maximizing therapeutic benefits and minimizing adverse effects. The successful implementation of AI/ML models in clinical practice could have significant implications for the management of NSCLC patients receiving immunotherapy. These models could aid in patient stratification and selection for immunotherapy, potentially improving treatment outcomes and reducing unnecessary toxicities and healthcare costs associated with ineffective treatments. Furthermore, AI/ML algorithms could be integrated into clinical decision support systems, providing real-time predictions and recommendations to oncologists based on patient-specific data. This could facilitate more informed and personalized treatment decisions, ultimately improving the quality of care and patient outcomes. Additionally, AI/ML models could be employed for continuous monitoring of treatment response, enabling timely adjustments to therapy based on individual patient trajectories. This approach aligns with the paradigm shift toward precision oncology, where treatment strategies are dynamically adapted based on real-time patient data and response patterns. However, it is crucial to note that the adoption of AI/ML technologies in clinical practice should be accompanied by robust validation, regulatory oversight, and ethical considerations. Moreover, healthcare professionals should receive proper training and education to ensure the effective and responsible utilization of these technologies.

## Conclusions

This systematic review highlights the promising potential of AI and ML techniques in predicting the response to immunotherapy in NSCLC. The included studies demonstrated the ability of these models to integrate diverse data sources, including medical imaging, genomic data, clinical variables, and immunohistochemical markers, to accurately predict immunotherapy response, PFS, and OS. However, challenges remain in the data availability, quality, and interpretability of these models, as well as the translation of these models from research settings to clinical practice. Future research should focus on developing interpretable and explainable AI/ML techniques, addressing ethical concerns, and ensuring the generalizability and robustness of these models across diverse patient populations and healthcare settings. Additionally, the integration of AI/ML models with traditional biomarkers and the exploration of novel algorithms and architectures could further enhance their predictive performance and clinical utility. Ultimately, the successful implementation of AI/ML models in clinical practice could enable personalized treatment strategies, improve patient outcomes, and reduce healthcare costs associated with ineffective treatments.
